# Prediction of fellow eye neovascularization in type 3 macular neovascularization (Retinal angiomatous proliferation) using deep learning

**DOI:** 10.1371/journal.pone.0310097

**Published:** 2024-10-30

**Authors:** Won Tae Yoon, Seong Jae Lee, Jae Hee Jeong, Jae Hui Kim

**Affiliations:** 1 Department of Ophthalmology, Kim’s Eye Hospital, Seoul, South Korea; 2 Kim’s Eye Hospital Data Center, Seoul, South Korea; 3 HumanDeep Inc., Seongnam-si, Gyeonggi-do, South Korea; Eye Foundation Hospital / Eye Foundation Retina Institute, NIGERIA

## Abstract

**Purpose:**

To establish a deep learning artificial intelligence model to predict the risk of long-term fellow eye neovascularization in unilateral type 3 macular neovascularization (MNV).

**Methods:**

This retrospective study included 217 patients (199 in the training/validation of the AI model and 18 in the testing set) with a diagnosis of unilateral type 3 MNV. The purpose of the AI model was to predict fellow eye neovascularization within 24 months after the initial diagnosis. The data used to train the AI model included a baseline fundus image and horizontal/vertical cross-hair scan optical coherence tomography images in the fellow eye. The neural network of this study for AI-learning was based on the visual geometry group with modification. The precision, recall, accuracy, and the area under the curve values of receiver operating characteristics (AUCROC) were calculated for the AI model. The accuracy of an experienced (examiner 1) and less experienced (examiner 2) human examiner was also evaluated.

**Results:**

The incidence of fellow eye neovascularization over 24 months was 28.6% in the training/validation set and 38.9% in the testing set (P = 0.361). In the AI model, precision was 0.562, recall was 0.714, accuracy was 0.667, and the AUCROC was 0.675. The sensitivity, specificity, and accuracy were 0.429, 0.727, and 0.611, respectively, for examiner 1, and 0.143, 0.636, and 0.444, respectively, for examiner 2.

**Conclusions:**

This is the first AI study focusing on the clinical course of type 3 MNV. While our AI model exhibited accuracy comparable to that of human examiners, overall accuracy was not high. This may partly be a result of the relatively small number of patients used for AI training, suggesting the need for future multi-center studies to improve the accuracy of the model.

## Introduction

Type 3 macular neovascularization (MNV) [1], also called retinal angiomatous proliferation [2] is a subtype of neovascular age-related macular degeneration (AMD) which constitutes 4.5% to 15% of all neovascular AMDs [3–5].

One of the distinct characteristics of type 3 MNV is the high risk of bilateral involvement [2, 6]. In particular, 38.3% to 100% of patients with unilateral type 3 MNV eventually experience fellow eye neovascularization during follow-up [7, 8]. It is well-known that several factors, including the presence of drusen and reticular pseudodrusen [9, 10], double layer sign, and hyperreflective foci [11] are associated with a risk of fellow eye neovascularization in neovascular AMD. However, to date, risk factors specific to type 3 MNV have not yet been fully elucidated.

Since fellow eye neovascularization may lead to bilateral visual deterioration [12], close follow-up of fellow eyes with detailed retinal examination is required to avoid treatment delay in type 3 MNV [13]. However, frequent hospital visits inevitably imposes a substantial time and financial burden on patients [14]. Therefore, accurate prediction of the risk of fellow eye neovascularization would be of considerable value in clinical care because the follow-up frequency could be adjusted based on the predicted risk for each patient.

Currently, artificial intelligence (AI) is widely utilized in AMD studies [15, 16]. Several articles demonstrated the performance and usefulness of AI models in predicting neovascular change in AMD [17–19]. To date, however, no previous study has specifically focused on type 3 MNV despite the fact that predicting fellow eye neovascularization is crucial in this disease subtype.

The purpose of the present study was to establish a deep learning AI model to predict the risk of long-term fellow eye neovascularization in unilateral type 3 MNV.

## Materials and methods

This retrospective observational study was conducted at a single center (Kim’s Eye Hospital, Seoul, South Korea). The study was approved by the Institutional Review Board of Kim’s Eye Hospital (#2022-04-016) and conducted in accordance with the tenets of the Declaration of Helsinki. Due to the retrospective nature of this study, the requirement for patient informed consent was waived (Kim’s Eye Hospital IRB, Seoul, South Korea). The data access dates for research purposes were February 3, 2023. Throughout the data collection process, no information that could identify individual participants was accessed.

### Study participants and treatment

This study included treatment-naïve patients diagnosed with type 3 MNV between January 2013 and March 2021 and initially treated with three loading injections of anti-vascular endothelial growth factor (anti-VEGF). The exclusion criteria were as follows: 1) less than 24 months of follow-up, 2) presence of definite chorioretinal anastomosis on fundus photography at diagnosis, 3) previous history of vitreoretinal surgery or glaucoma surgery, and 4) low OCT image quality. When both eyes met the inclusion criteria, the eyes which were affected first were enrolled in the study.

At the initial diagnosis, all patients underwent fundus photography (Canon CX-1, Tokyo, Japan), fluorescein angiography, indocyanine-green angiography, and optical coherence tomography (OCT) examinations. All the OCT scans were performed using Spectralis^®^ device (Heidelberg Engineering, Heidelberg, Germany). The diagnosis of type 3 MNV was made when characteristic intraretinal lesions with surrounding retinal edema were identified on optical coherence tomography (OCT), accompanied by focal hyperfluorescence observed on angiography at the location of the lesion [20].

All patients underwent three monthly injections of ranibizumab (0.5 mg/0.05 mL of Lucentis^®^; Genentech Inc., San Francisco, CA, USA) or aflibercept (2.0 mg/0.05 mL of Eylea^®^; Regeneron, Tarrytown, NY, USA) in the involved eye. After the initial treatment, the fellow eye was examined at intervals of 2 to 6 months, as determined by the attending doctor. Fundus photo and OCT images of the fellow eye were taken at each follow-up visit.

The purpose of the AI model was to predict fellow eye neovascularization within 24 months of initial diagnosis. The following data were collected for AI training: patient age, sex, occurrence of fellow eye neovascularization, and the timing of fellow eye neovascularization. Fellow-eye fundus photographs and OCT images at the time of diagnosis were collected from all patients (These data were used for AI model training to predict fellow-eye neovascularization). For patients with fellow-eye neovascularization, fundus photos and OCT images at the time of neovascularization occurrence were additionally collected (This additional data was used for AI model training to predict OCT images of fellow-eye neovascularization).

### Data preprocessing

**Fig 1:**
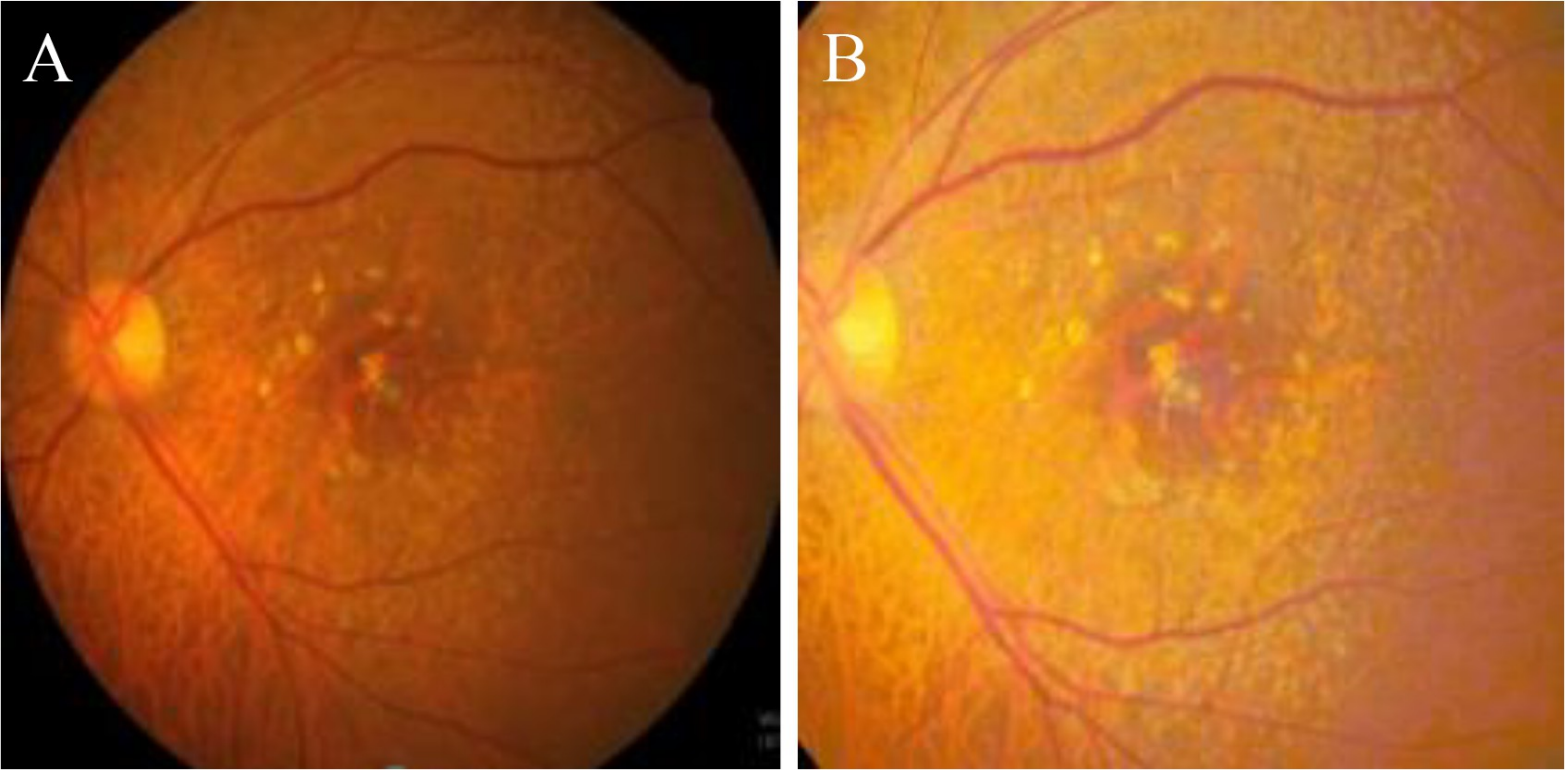
Fundus image before (left) and after preprocessing (right).

The data used to train the fellow eye neovascularization prediction model included a baseline fundus image and horizontal/vertical cross-hair scan OCT images in the fellow eye. Two data preprocessing techniques were performed for smooth training and performance improvement. In the first step in data preprocessing, background crop and resize were performed on the fundus and OCT images. The second step in data preprocessing was performed to improve the color of the fundus image and histogram equalization [21] was applied to better identify drusen and pseudodrusen (Fig 1).

### Model architecture

**Fig 2:**
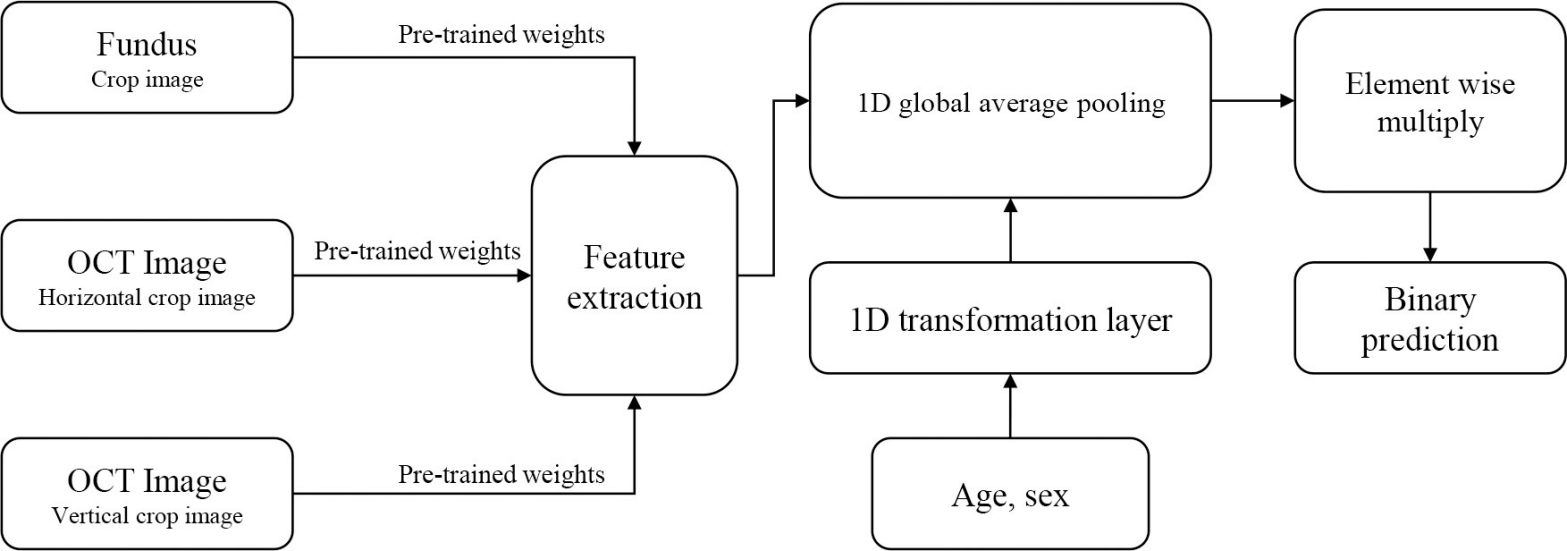
The structure of the artificial intelligence model with three input paths which utilizes data on age and sex and ultimately combines the data through element-specific multiplication methods. Abbreviations: OCT = optical coherence tomography, D = dimension.

The neural network of this study is a convolutional neural network-based multi-input network with three input paths to process pre-processed fundus and light interference tomography images at the same time, in which each network path was used by modifying the visual geometry group (VGG) [22] network. Based on this multi input feature extraction network, we used a multimodal network that additionally utilizes data on age and sex. The entire network consists of a part that simultaneously receives three images as input and extracts features, and a part that converts data on age and sex into vectors. The features derived from each part are as follows: a one-dimensional vector through element-specific multiplication and combination methods, a fully connected layer and dropout, and a classifier for binary classification. (Fig 2).

### Data augmentation and transfer learning for each path

We trained the model using data augmentation and transfer learning [23]. In this case, data augmentation uses random vertical and horizontal flip along with random rotation. Color conversion enhancement such as Jitter, which is commonly used in image data enhancement, was not applied as this decreased model generalization performance. In addition, transfer learning was performed to improve the stability and performance of multimodal networks trained by receiving three images, and transfer learning was trained by organizing fundus and OCT images into a single network instead of commonly used ImageNet weights. The structure and training methods of a single network for transfer learning were configured to have the same configuration as all methods used to train this network.

### Other training strategy

Additional strategies applied to improve AI training are as follows. The class weight was applied to match the positive/negative ratio of all data, and the learning rate was reduced by 8% per 8 epoch for smooth training of the network as learning progressed. In addition, early stopping was applied to prevent overfitting that occurs during learning and to find points with optimal weights. Finally, L2 regularization (5e-4) was applied to the entire convolutional layer. This, along with dropouts, applied large values to maximally suppress overfitting of the entire network. Training was conducted using a batch size of 4 with Adam optimizer with an initial learning rate of 5e-4.

### Evaluation of performance of the AI model in predicting the development of fellow eye neovascularization

The entire network was trained and verified through 5-fold cross validation because the amount of data available for learning was small. The verification performance was then measured by averaging both the F1 score and the Area Under the Curve (AUC) values of Receiver Operating Characteristics (ROC) derived from each fold. In this case, each indicator was measured and evaluated based on the optimal cut-off value that maximizes the corresponding score.

### Comparison of performance to predict fellow eye neovascularization between AI model and human examiners

Testing for performance of the AI model was planned using horizonal and vertical cross-hair OCT images from 18 patients who were not included in the AI model training. The performance of AI model was assessed by two retina specialists with different degrees of experience (experienced: J.H.K., examiner 1; less experienced: W.T.Y., examiner 2). The sensitivity, specificity, and accuracy of predicting the fellow eye neovascularization were calculated. In addition, the area under the curve (AUC) values of the receiver operating characteristics (ROC) curve were calculated.

### Performance of the AI model in predicting OCT images when the fellow eye neovascularization has developed

The AttentionGAN [AttentionGAN: Unpaired Image-to-Image Translation Using Attention-Guided Generative Adversarial Networks] algorithm [24] was adopted in the OCT image synthesis of the fellow eye neovascularization. AttentionGAN was utilized for synthesizing OCT images of fellow eye neovascularization development. The AI-generated OCT images were reviewed to identify the presence of intraretinal fluid (IRF) and pigment epithelial detachment (PED). According to the previously suggested classification [25], the presence of IRF only was classified as stage 2 and the presence of PED was classified as stage 3. The AI-generated OCT images were used for classification, and their accuracy was compared with the classification based on actual images.

### Statistical analysis

Data are presented as the mean ± standard deviation or numbers (%), where applicable. Differences in characteristics between the training set and the testing set were compared using the Mann-Whitney *U* test, chi-square test, or Fisher’s exact test. In this analysis, Statistical Package for the Social Sciences for Windows^®^ (version 21.0; IBM, Armonk, NY, USA) was used and statistical significance was set at P < 0.05. AUC values of the ROC curve were calculated using Scikit-learn.

## Results

A total of 217 patients participated in this study. Of these, 159 participated in training the AI model, and 40 participated in the validation set. The remaining 18 patients were included in the AI model test. Of the 159 eyes included in the training set, 41 (25.8%) were in stage 2 and 118 (74.2%) were in stage 3. The mean fellow-eye examination interval was 4.6 ± 0.5 months. When comparing the training and testing sets (Table 1), there were no differences in age (P = 0.321), sex (P = 0.355), diabetes mellitus (P = 0.868), hypertension (P = 0.809), or incidence of fellow eye neovascularization within 24 months (P = 0.361). The timing of fellow-eye neovascularization in the training set was 13.7 ± 6.3 months after diagnosis.

**Table 1:** Characteristics of patients included in the training/validation and testing sets.

Characteristics	Training and validation set (n = 199)	Testing set (n = 18)	*p*-value
Age	75.86 ± 6.34	74.28 ± 7.78	0.321*
Sex			0.355†
Men	26 (13.1%)	1 (5.6%)	
Women	173 (86.9%)	17 (94.4%)	
DM	59(29.6%)	5(27.8%)	0.868†
HTN	127(63.8%)	12(66.7%)	0.809†
Incidence of fellow eye neovascularization	57(28.6%)	8(38.9%)	0.361†

Data are presented as mean ± standard deviation or No.(%) where applicable

*Independent sample t-test.

† Chi-square test.

### Performance of the AI model in predicting the development of fellow eye neovascularization

**Fig 3:**
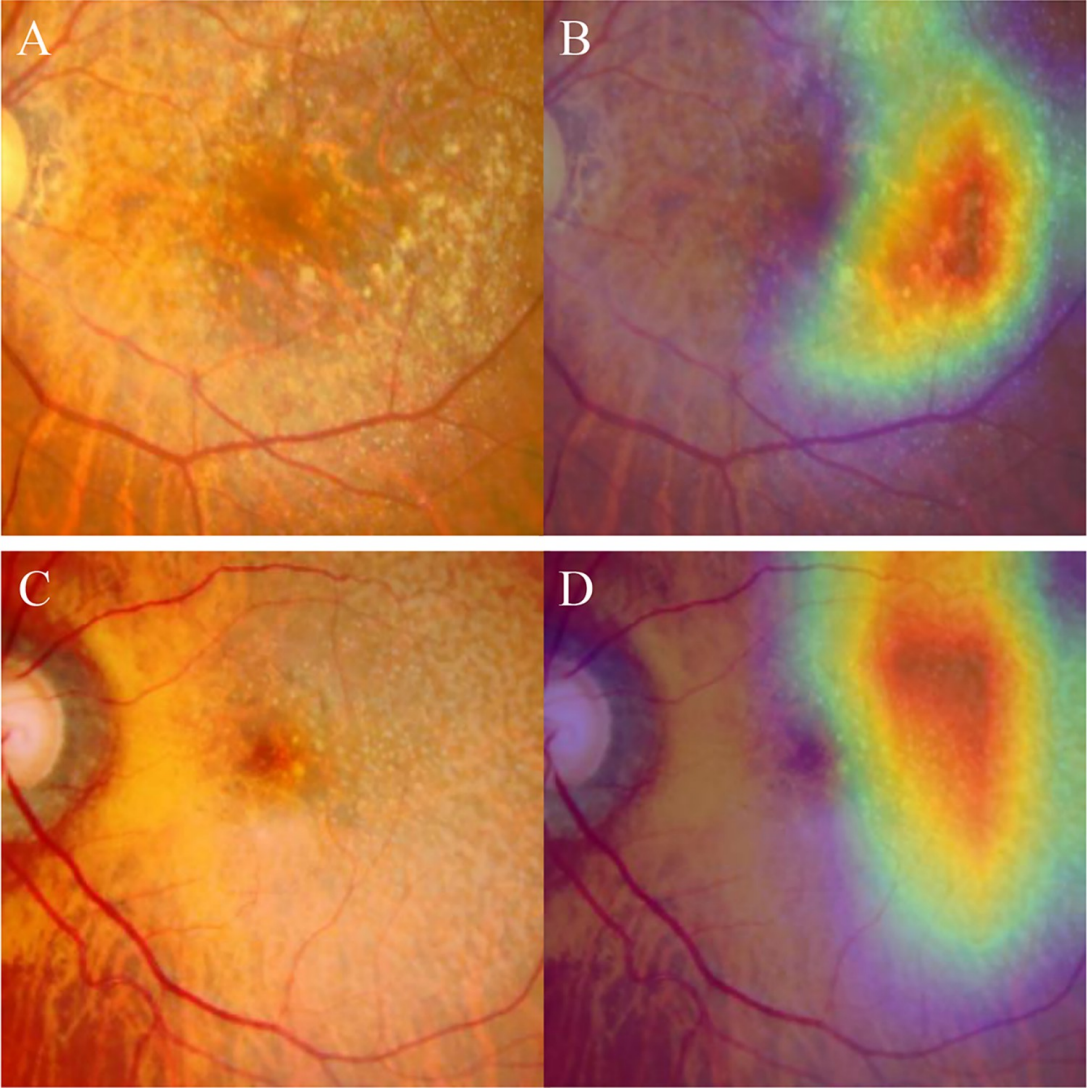
Representative original fundus photos (A,C) and corresponding heatmaps (B,D). Note that the artificial intelligence prioritizes the region temporal to the fovea.

The 5-fold cross validation was performed for data from 159 patients included in the training and validation set (Table 2). The F1 scores derived from each subset were between 0.511–0.779 and the AUCROC scores derived from each subset were between 0.516–0.785. The mean F1 score was 0.624 and the mean AUCROC score was 0.668. A representative heatmap image is shown in Fig 3.

**Table 2:** Results of 5-fold cross validation.

Subset	F1 score	AUCROC score
1 Fold (n = 159)	0.619	0.683
2 Fold (n = 159)	0.643	0.752
3 Fold (n = 159)	0.779	0.785
4 Fold (n = 159)	0.568	0.601
5 Fold (n = 159)	0.511	0.516
Result	0.624	0.668

Abbreviations: AUCROC = Area Under the Curve values of Receiver Operating Characteristics

**Fig 4:**
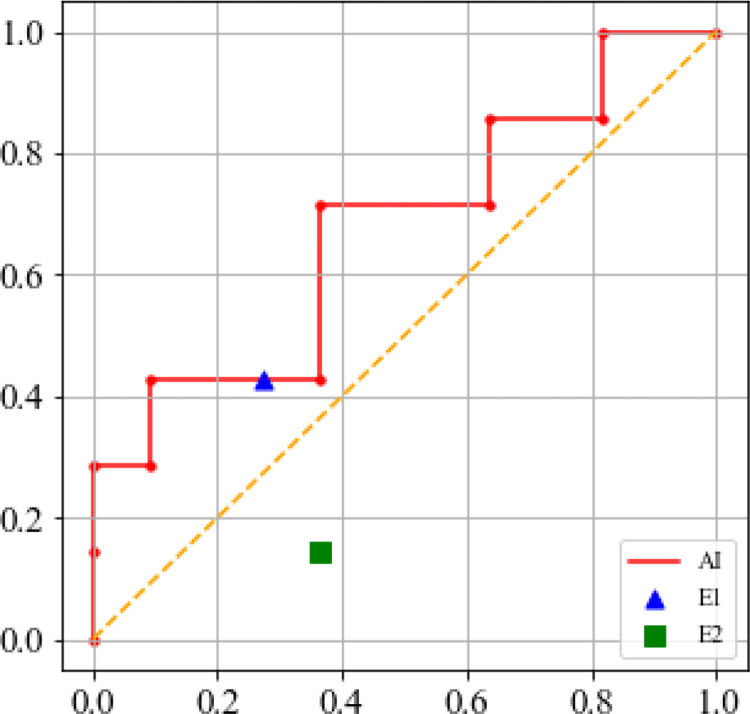
Ensemble performance of the artificial intelligence model in predicting the incidence of fellow eye neovascularization. The outcomes from 5-fold cross validation were used to calculate the AUC and F1 score. The precision was 0.562, recall was 0.714, accuracy was 0.667, weighted F1 score was 0.671, AUCROC was 0.675. E1 = examiner 1 (experienced examiner), E2 = examiner 2 (less experienced examiner).

The ensemble performance of the AI model in predicting the incidence of fellow eye neovascularization is presented in Fig 4. The precision was 0.562, recall was 0.714, accuracy was 0.667, weighted F1 score was 0.671, AUCROC is 0.675. The sensitivity, specificity, and accuracy were 0.429, 0.727, and 0.611, respectively, for examiner 1 and 0.143, 0.636, and 0.444, respectively, for examiner 2. The AI model shows a comparable performance to the experienced examiner and shows a relatively higher performance than that recorded for the less experienced examiner.

### Performance of the AI model in predicting OCT images when the fellow eye neovascularization has developed

**Fig 5:**
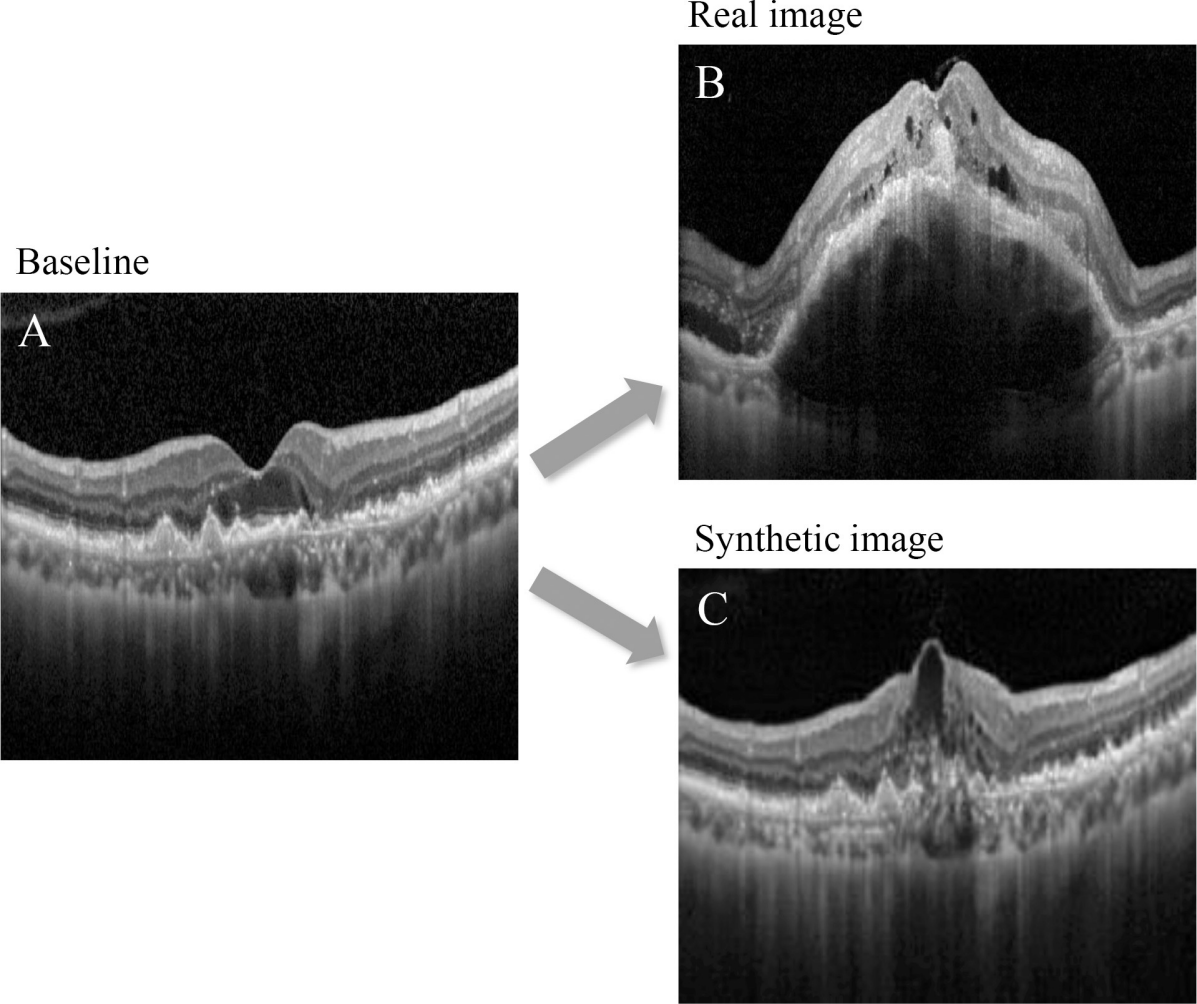
A representative case illustrating stage 3 fellow eye neovascularization mis-predicted as stage 2 in the synthesized image using artificial intelligence. A: real optical coherence tomography image at baseline, B: real image when the fellow eye neovascularization was noted, C: artificial intelligence-synthesized image of fellow eye neovascularization. While the synthetic image demonstrates the occurrence of intraretinal fluid, it was unable to accurately predict the development of pigment epithelial detachment.

Eighteen cases of fellow eye neovascularization were used for this analysis. When fellow eye neovascularization was first noted, 6 cases were classified as stage 2 and 12 cases were classified as stage 3. When using the AI-generated images for predictions, out of 6 actual stage 2 cases, 5 were correctly predicted as stage 2, but one was misclassified as stage 3. Among the actual stage 3 cases, three were correctly predicted as stage 3, but nine were incorrectly classified as stage 2. Fig 5 illustrates the differences between the OCT images synthesized by the AI model and actual images.

## Discussion

In unilateral type 3 MNV, the fellow eye is at high risk of progression to late stage AMD, including both neovascularization and retinal pigment epithelial (RPE) atrophy. Kim et al., reported neovascularization in 50.9% of the fellow eyes and RPE atrophy without neovascularization in 10.8% of fellow eyes over a mean follow-up period of 45.9 months [12]. This result suggests that long-term good visual acuity in the fellow eye may not be guaranteed in patients with unilateral type 3 MNV.

There is no consensus on the optimal fellow eye examination interval in patients with unilateral type 3 MNV. The study by Kim et al., had a mean fellow eye examination interval of 4.8 ± 2.2 months (range, 2 to 10 months) [13]. In that study, a longer fellow eye examination interval was associated with poor visual acuity and greater visual deterioration of the fellow eye with neovascularization [13], underscoring the need for frequent monitoring of the fellow eye.

Typically, considerable time is required for a patient with neovascular AMD to visit the hospital and undergo examination [14]. Furthermore, providing patients with transportation to appointments often requires caregivers to also take time away from work and personal activities [14]. Accurate prediction of the risk of fellow eye neovascularization would be helpful to set up a more efficient hospital visit schedule. The presence of reticular pseudodrusen in the fellow eye was found to be a biomarker that is closely associated with the risk of fellow eye neovascularization in unilateral type 3 MNV [7, 26, 27]. However, since reticular pseudodrusenis a frequently reported finding in type 3 MNV [12], reticular pseudodrusen alone is often not sufficient to adequately assess the risk of neovascularization. Currently, accurately predicting fellow eye neovascularization in type MNV poses a significant challenge, even for experienced clinicians.

Previous studies have shown the potential of an AI model to predict the neovascularization in AMD. Yim et al. attempted to predict the risk of conversion to neovascular AMD within the next 6 months based on data from 386 patients [17]. The performance of the AI model was better than that recorded for 5 out of 6 experts, with a sensitivity of 80%, specificity of 55%, and AUC of 0.765 [17]. Banerjee et al. used a deep sequence model to predict fellow eye exudation based on data from 671 patients [19]. The AUC values were 0.82 for the prediction of exudation within 3 months and 0.68 for the prediction of exudation within 21 months [19]. In a more recent study, Ajana et al., predicted the long-term progression to advanced AMD based on data from 3,838 patients with early or intermediate AMD [18]. The AUC was 0.92 at 5 years, 0.92 at 10 years, and 0.91 at 15 years [18].

In the present study, we attempted to predict fellow eye neovascularization in unilateral type 3 MNV based on an AI-model. As far as we are aware, this is the first AI study focusing on type 3 MNV. While our AI model exhibited accuracy that was not inferior to that recorded for the two human examiners, the overall accuracy of the model was not high. We consider that the primary reason for this outcome is the difficulty of the prediction itself. Even the experienced examiner achieved an accuracy of only 0.611, demonstrating the inherent difficulty in predicting fellow eye neovascularization in unilateral type 3 MNV. The relatively small number of patients used for AI learning is another relevant reason for our findings. Enrolling adequate numbers of patients is challenging because type 3 MNV constitutes only a small proportion of the overall neovascular AMD. Therefore, further studies with a larger dataset are required to improve the accuracy of AI models. Discussions are currently underway to establish an AI model that can be utilized in ophthalmology practice [15, 28]. Collaboration among various centers is crucial to gather sufficient data for conditions with a low incidence, such as type 3 MNV.

Upon inspecting the heatmap images, it was observed that the AI model placed significant importance on the area surrounding the foveal center rather than directly on the foveal center in fundus photos. It is worth noting that the locations prioritized by the AI model closely corresponded to the distribution of reticular pseudodrusen. It is well-known that the distribution of reticular pseudodrusen is predominantly characterized by sparing of the fovea [29, 30]. It was postulated that this distinctive distribution was one of the potential reasons contributing to the fovea-sparing developmental pattern observed in type 3 MNV [31]. The exact reason for this phenomenon remains undisclosed within the black box, and it is uncertain whether the AI model recognized the distribution of reticular pseudodrusen and utilized it to predict the occurrence of fellow eye neovascularization. We anticipate that future advancements in AI algorithms for automatic detection of reticular pseudodrusen [32] will potentially enhance the predictive performance of fellow eye neovascularization in type 3 MNV.

In the present study, we attempted to predict the stage of fellow eye neovascularization at the time of its occurrence. In type 3 MNV, identifying the disease stage is important because it is closely associated with prognosis [33]. Our results show that predictions using AI-generated OCT images were useful in predicting the occurrence of stage 2 disease, although they did not show satisfactory performance in predicting the occurrence of stage 3 disease. These findings are likely attributed to the relatively limited number of images used for AI training in our study, suggesting that further research is needed to enhance predictive capabilities.

Generally, the test set comprised approximately 10–20% of the total dataset. Because the number of patients in our study was relatively small, including too many patients in the test set could reduce the number of patients in the training set, potentially affecting AI performance. Therefore, the test set was set to 10%. If the study population increases, the proportion of participants used for validation can also increase.

In predicting the OCT appearance of fellow eye neovascularization using AI, generating the shape of the PED on OCT is necessary to predict the form of stage 3 disease. The poor performance in predicting stage 3 suggests that AI often fails to generate PED when predicting the appearance of fellow eye neovascularization. The exact reason for this trend is unclear; however, one possible reason is the insufficient number of images used for training.

AI has been studied for various aspects of AMD. Notably, AI is known to perform well in diagnosing AMD using fundus photographs or OCT images [34, 35], suggesting that it could be effectively utilized in AMD screening [36]. With an aging global population, the number of patients with AMD is expected to continue increasing [37], underscoring the growing importance of early AMD diagnosis. Consequently, the role of AI in AMD screening is expected to become increasingly significant.

The strength of the present study is that, to the best of our knowledge, this is the first attempt to develop an AI model for predicting fellow eye neovascularization in unilateral type 3 MNV. However, this study has several limitations. First, it was a retrospective study performed at a single center. Second, only horizontal and vertical OCT images were analyzed. Because type 3 MNV lesions typically exhibit a fovea-sparing distribution [31], using only cross-hair OCT scans in AI learning may not be sufficient to predict the development or the OCT features of fellow-eye neovascularization. Further studies using raster-scan images covering the entire macular area are necessary to improve the prediction of neovascularization in the fellow eye. Third, data from a small number of patients were used for training, which may have affected the performance of the AI model. Therefore, the results of this study are not conclusive and are subject to validation in studies with larger sample sizes. Considering the low incidence of type 3 MNV, further multicenter studies are required to overcome these limitations. Fourth, the mean period of fellow-eye neovascularization is reported to be between 22.7 to 30 months [12, 26]. Nevertheless, in the present study, we analyzed follow-up data up to 24 months. The follow-up period was limited to 24 months because, at our institution, the follow-up loss rate tended to increase after this point. Future studies using long-term follow-up data are necessary to establish a model that can predict long-term outcomes. Fifth, OCT-angiography, a widely adopted examination method for identifying the characteristics of neovascularization, was not performed. Sixth, only a small number of participants were included in the test set. Finally, all patients were Korean; therefore, the generalizability of our results to other ethnic groups needs to be approached with caution.

In conclusion, our study demonstrated that an AI model utilizing deep learning can predict fellow eye neovascularization in type 3 MNV with an accuracy comparable to that of human examiners. Although our study has clear limitations, it is the first investigation utilizing AI to explore approaches to alleviate the burden on patients with type 3 MNV.

## Supporting information

S1 Checklist STROBE statement—checklist of items that should be included in reports of observational studies. (DOCX)

S1 Data (SAV)
