# Supplementary material for: Prediction of fellow eye neovascularization in type 3 macular neovascularization (Retinal angiomatous proliferation) using deep learning
Source: PLoS One. 2024 Oct 30;19(10):e0310097. doi: 10.1371/journal.pone.0310097 (PMC11524474; doi:10.1371/journal.pone.0310097)
Supplement: S1 Checklist — (DOCX) [file pone.0310097.s001.docx]

STROBE Statement—checklist of items that should be included in reports of observational studies

|  | Item No. | Recommendation | Page  No. | Relevant text from manuscript |
| --- | --- | --- | --- | --- |
| **Title and abstract** | 1 | (*a*) Indicate the study’s design with a commonly used term in the title or the abstract | 1 | Prediction of Fellow Eye Neovascularization in Type 3 Macular Neovascularization (Retinal Angiomatous Proliferation) using Deep Learning |
|  |  | (*b*) Provide in the abstract an informative and balanced summary of what was done and what was found | 2 | **Purpose:** To establish a deep learning artificial intelligence model to predict the risk of long-term fellow eye neovascularization in unilateral type 3 macular neovascularization (MNV).  **Methods:** This retrospective study included 217 patients (199 in the training/validation of the AI model and 18 in the testing set) with a diagnosis of unilateral type 3 MNV. The purpose of the AI model was to predict fellow eye neovascularization within 24 months after the initial diagnosis. The data used to train the AI model included a baseline fundus image and horizontal/vertical cross-hair scan optical coherence tomography images in the fellow eye. The neural network of this study for AI-learning was based on the visual geometry group with modification. The precision, recall, accuracy, and the area under the curve values of receiver operating characteristics (AUCROC) were calculated for the AI model. The accuracy of an experienced (examiner 1) and less experienced (examiner 2) human examiner was also evaluated.  **Results:** The incidence of fellow eye neovascularization over 24 months was 44.2% in the training/validation set and 38.9% in the testing set (P = 0.662). In the AI model, precision was 0.562, recall was 0.714, accuracy was 0.667, and the AUCROC was 0.675. The sensitivity, specificity, and accuracy were 0.429, 0.727, and 0.611, respectively, for examiner 1, and 0.143, 0.636, and 0.444, respectively, for examiner 2.  **Conclusions:** This is the first AI study focusing on the clinical course of type 3 MNV. While our AI model exhibited accuracy comparable to that of human examiners, overall accuracy was not high. This may partly be a result of the relatively small number of patients used for AI training, suggesting the need for future multi-center studies to improve the accuracy of the model. |
| Introduction | | | |  |
| Background/rationale | 2 | Explain the scientific background and rationale for the investigation being reported | 3 | Type 3 macular neovascularization (MNV), also called retinal angiomatous proliferation is a subtype of neovascular age-related macular degeneration (AMD) which constitutes 4.5% to 15% of all neovascular AMDs. One of the distinct characteristics of type 3 MNV is the high risk of bilateral involvement. In particular, 38.3% to 100% of patients with unilateral type 3 MNV eventually experience fellow eye neovascularization during follow-up. It is well-known that several factors, including the presence of drusen and reticular pseudodrusen, double layer sign, and hyperreflective foci are associated with a risk of fellow eye neovascularization in neovascular AMD. However, to date, risk factors specific to type 3 MNV have not yet been fully elucidated.  Since fellow eye neovascularization may lead to bilateral visual deterioration, close follow-up of fellow eyes with detailed retinal examination is required to avoid treatment delay in type 3 MNV. However, frequent hospital visits inevitably imposes a substantial time and financial burden on patients. Therefore, accurate prediction of the risk of fellow eye neovascularization would be of considerable value in clinical care because the follow-up frequency could be adjusted based on the predicted risk for each patient. Currently, artificial intelligence (AI) is widely utilized in AMD studies. Several articles demonstrated the performance and usefulness of AI models in predicting neovascular change in AMD. To date, however, no previous study has specifically focused on type 3 MNV despite the fact that predicting fellow eye neovascularization is crucial in this disease subtype. As the incidence of RVO is lower in younger adults, information regarding the risk factors of RVO in the younger population is limited. |
| Objectives | 3 | State specific objectives, including any prespecified hypotheses | 3 | The purpose of the present study was to establish a deep learning AI model to predict the risk of long-term fellow eye neovascularization in unilateral type 3 MNV. |
| Methods | | | |  |
| Study design | 4 | Present key elements of study design early in the paper | 3 | Retrospective observational study |
| Setting | 5 | Describe the setting, locations, and relevant dates, including periods of recruitment, exposure, follow-up, and data collection | 3-4 | This retrospective observational study included treatment-naïve patients diagnosed with type 3 MNV between January 2013 and March 2021 and initially treated with three loading injections of anti-vascular endothelial growth factor (anti-VEGF). |
| Participants | 6 | (*a*) *Cohort study*—Give the eligibility criteria, and the sources and methods of selection of participants. Describe methods of follow-up  *Case-control study*—Give the eligibility criteria, and the sources and methods of case ascertainment and control selection. Give the rationale for the choice of cases and controls  *Cross-sectional study*—Give the eligibility criteria, and the sources and methods of selection of participants | 4 | This study included treatment-naïve patients diagnosed with type 3 MNV between January 2013 and March 2021 and initially treated with three loading injections of anti-vascular endothelial growth factor (anti-VEGF). The exclusion criteria were as follows: 1) less than 24 months of follow-up, 2) presence of definite chorioretinal anastomosis on fundus photography at diagnosis, 3) previous history of vitreoretinal surgery or glaucoma surgery, and 4) low OCT image quality. When both eyes met the inclusion criteria, the eyes which were affected first were enrolled in the study. |
|  |  | (*b*) *Cohort study*—For matched studies, give matching criteria and number of exposed and unexposed  *Case-control study*—For matched studies, give matching criteria and the number of controls per case | NA |  |
| Variables | 7 | Clearly define all outcomes, exposures, predictors, potential confounders, and effect modifiers. Give diagnostic criteria, if applicable | 4 |  |
| Data sources/ measurement | 8* | For each variable of interest, give sources of data and details of methods of assessment (measurement). Describe comparability of assessment methods if there is more than one group | *4* | The following data were collected for AI training: patient age, sex, occurrence of fellow eye neovascularization, timing of fellow eye neovascularization, and fundus photographs and OCT images in the fellow eye taken at the time of diagnosis and when fellow eye neovascularization was detected. |
| Bias | 9 | Describe any efforts to address potential sources of bias |  |  |
| Study size | 10 | Explain how the study size was arrived at | 7 | A total of 217 patients participated in the study. |

Continued on next page

| Quantitative variables | 11 | Explain how quantitative variables were handled in the analyses. If applicable, describe which groupings were chosen and why | 6-7 | We trained the model using data augmentation and transfer learning. In this case, data augmentation uses random vertical and horizontal flip along with random rotation. Color conversion enhancement such as Jitter, which is commonly used in image data enhancement, was not applied as this decreased model generalization performance. In addition, transfer learning was performed to improve the stability and performance of multimodal networks trained by receiving three images, and transfer learning was trained by organizing fundus and OCT images into a single network instead of commonly used ImageNet weights. The structure and training methods of a single network for transfer learning were configured to have the same configuration as all methods used to train this network. |
| --- | --- | --- | --- | --- |
| Statistical methods | 12 | (*a*) Describe all statistical methods, including those used to control for confounding | 6-7 | The entire network was trained and verified through 5-fold cross validation because the amount of data available for learning was small. The verification performance was then measured by averaging both the F1 score and the Area Under the Curve (AUC) values of Receiver Operating Characteristics (ROC) derived from each fold. In this case, each indicator was measured and evaluated based on the optimal cut-off value that maximizes the corresponding score.  Testing for performance of the AI model was planned using horizonal and vertical cross-hair OCT images from 18 patients who were not included in the AI model training. The performance of AI model was assessed by two retina specialists with different degrees of experience (experienced: J.H.K., examiner 1; less experienced: W.T.Y., examiner 2). The sensitivity, specificity, and accuracy of predicting the fellow eye neovascularization were calculated. In addition, the area under the curve (AUC) values of the receiver operating characteristics (ROC) curve were calculated.  Data are presented as the mean ± standard deviation or numbers (%), where applicable. Differences in characteristics between the training set and the testing set were compared using the Mann-Whitney *U* test, chi-square test, or Fisher’s exact test. In this analysis, Statistical Package for the Social Sciences for Windows^®^ (version 21.0; IBM, Armonk, NY, USA) was used and statistical significance was set at P < 0.05. AUC values of the ROC curve were calculated using Scikit-learn. |
|  |  | (*b*) Describe any methods used to examine subgroups and interactions | NA |  |
|  |  | (*c*) Explain how missing data were addressed |  |  |
|  |  | (*d*) *Cohort study*—If applicable, explain how loss to follow-up was addressed  *Case-control study*—If applicable, explain how matching of cases and controls was addressed  *Cross-sectional study*—If applicable, describe analytical methods taking account of sampling strategy | NA |  |
|  |  | (*e*) Describe any sensitivity analyses | NA |  |
| Results | | | | |
| Participants | 13* | (a) Report numbers of individuals at each stage of study—eg numbers potentially eligible, examined for eligibility, confirmed eligible, included in the study, completing follow-up, and analysed | 7 | A total of 217 patients participated in the study. Of these, 159 participated in the training of the AI model and 40 participated in the validation set. The remaining 18 patients participated in the test of the AI model. |
|  |  | (b) Give reasons for non-participation at each stage | NA |  |
|  |  | (c) Consider use of a flow diagram | NA |  |
| Descriptive data | 14* | (a) Give characteristics of study participants (eg demographic, clinical, social) and information on exposures and potential confounders | 7 | When comparing the training and testing sets (Table 1), there was no difference in age (P = 0.321), sex (P =0.355), diabetes mellitus (P =0.868), hypertension (P = 0.809), and the incidence of fellow eye neovascularization within 24 months (P = 0.662). |
|  |  | (b) Indicate number of participants with missing data for each variable of interest | NA |  |
|  |  | (c) *Cohort study*—Summarise follow-up time (eg, average and total amount) | NA |  |
| Outcome data | 15* | *Cohort study*—Report numbers of outcome events or summary measures over time | NA |  |
|  |  | *Case-control study—*Report numbers in each exposure category, or summary measures of exposure | NA |  |
|  |  | *Cross-sectional study—*Report numbers of outcome events or summary measures | NA |  |
| Main results | 16 | (*a*) Give unadjusted estimates and, if applicable, confounder-adjusted estimates and their precision (eg, 95% confidence interval). Make clear which confounders were adjusted for and why they were included | 9 | The ensemble performance of the AI model in predicting the incidence of fellow eye neovascularization is presented in Figure 4. The precision was 0.562, recall was 0.714, accuracy was 0.667, weighted F1 score was 0.671, AUCROC is 0.675. The sensitivity, specificity, and accuracy were 0.429, 0.727, and 0.611, respectively, for examiner 1 and 0.143, 0.636, and 0.444, respectively, for examiner 2. The AI model shows a comparable performance to the experienced examiner and shows a relatively higher performance than that recorded for the less experienced examiner. |
|  |  | (*b*) Report category boundaries when continuous variables were categorized | NA |  |
|  |  | (*c*) If relevant, consider translating estimates of relative risk into absolute risk for a meaningful time period | NA |  |

Continued on next page

| Other analyses | 17 | Report other analyses done—eg analyses of subgroups and interactions, and sensitivity analyses | 9 | Eighteen cases of fellow eye neovascularization were used for this analysis. When fellow eye neovascularization was first noted, 6 cases were classified as stage 2 and 12 cases were classified as stage 3. When using the AI-generated images for predictions, out of 6 actual stage 2 cases, 5 were correctly predicted as stage 2, but 1 case was misclassified as stage 3. Among the actual stage 3 cases, 3 were correctly predicted as stage 3, but 9 cases were incorrectly classified as stage 2. Figure 5 illustrates the differences between OCT images synthesized by the AI model and actual images |
| --- | --- | --- | --- | --- |
| Discussion | | | | |
| Key results | 18 | Summarise key results with reference to study objectives | 2 | The incidence of fellow eye neovascularization over 24 months was 44.2% in the training/validation set and 38.9% in the testing set (P = 0.662). In the AI model, precision was 0.562, recall was 0.714, accuracy was 0.667, and the AUCROC was 0.675. The sensitivity, specificity, and accuracy were 0.429, 0.727, and 0.611, respectively, for examiner 1, and 0.143, 0.636, and 0.444, respectively, for examiner 2.  This is the first AI study focusing on the clinical course of type 3 MNV. While our AI model exhibited accuracy comparable to that of human examiners, overall accuracy was not high. This may partly be a result of the relatively small number of patients used for AI training, suggesting the need for future multi-center studies to improve the accuracy of the model. |
| Limitations | 19 | Discuss limitations of the study, taking into account sources of potential bias or imprecision. Discuss both direction and magnitude of any potential bias | 12 | First, it is a retrospective study performed at a single center. Second, since only horizontal and vertical OCT scan images were used for the analysis, the outcome may differ when using all the raster scan images. Third, data from a small number of patients were used for training and this may influence the performance of the AI model. Considering the low incidence of type 3 MNV, further multicenter studies are required to overcome this shortcoming. Lastly, all the patients were Korean so the generalizability of our results to other ethnic groups needs to be approached with caution. |
| Interpretation | 20 | Give a cautious overall interpretation of results considering objectives, limitations, multiplicity of analyses, results from similar studies, and other relevant evidence | 12 |  |
| Generalisability | 21 | Discuss the generalisability (external validity) of the study results | NA |  |
| Other information | |  | | |
| Funding | 13 | Give the source of funding and the role of the funders for the present study and, if applicable, for the original study on which the present article is based | NA | This study was supported by Kim’s Eye Hospital Research Center. The funder had no role in study design, data collection and analysis, decision to publish. |

*Give information separately for cases and controls in case-control studies and, if applicable, for exposed and unexposed groups in cohort and cross-sectional studies.

**Note:** An Explanation and Elaboration article discusses each checklist item and gives methodological background and published examples of transparent reporting. The STROBE checklist is best used in conjunction with this article (freely available on the Web sites of PLoS Medicine at http://www.plosmedicine.org/, Annals of Internal Medicine at http://www.annals.org/, and Epidemiology at http://www.epidem.com/). Information on the STROBE Initiative is available at www.strobe-statement.org.
